# Statistical errors undermine claims about the evolution of polysynthetic languages

**DOI:** 10.1073/pnas.2518416122

**Published:** 2025-10-08

**Authors:** Alexander Koplenig, Sascha Wolfer

**Affiliations:** ^a^Department for Lexical Studies, Leibniz Institute for the German Language (IDS), Mannheim 68161, Germany

Bromham et al. (1) claim that polysynthetic languages tend to have smaller populations, fewer neighbouring languages in contact, and greater phylogenetic isolation. We show that the statistical analysis behind these claims contains key flaws that undermine the robustness of the results.

First, (1) claim to use Bayesian Model Averaging (BMA; see their table 2), but their code contains no Bayesian elements. BMA requires prior distributions over both models and parameters (2), yet (1) instead uses a form of frequentist model averaging (FMA), fitting all predictor combinations and using the maximum likelihood estimate (MLE) to compute posterior model probabilities (PMPs). This is incorrect (3) and is highly consequential: MLEs are not penalized for model complexity and lead to overfitting, as reflected in their table 2 where all posterior inclusion probabilities (PIPs) exceed 0.5. The appropriate FMA approximation to BMA uses the Bayesian Information Criterion (BIC) as PMP (3). We corrected the code and recomputed approximate PIPs without parameter averaging, which lacks justification in nonlinear models (4). Table 1 shows that only three variables (L1.pop, Bordering languages/km, and Altitude) consistently have PIPs > 0.5 across both Polysynthetic and Extended. Crucially, “Small Family”—a central finding in ref. 1—no longer meets this threshold (both PIPs < 0.06), calling into question the idea that polysynthetic languages tend to occur as “long, lonely lineages.”

**Table 1.** Corrected importance of variables for polysynthetic languages and the extended list

Variable	Polysynthetic (original)	Polysynthetic (BIC-based)	Extended (original)	Extended (BIC-based)
L1.pop	**1.000**	**1.000**	**1.000**	**1.000**
Area	**0.976**	0.292	**0.955**	0.051
Bordering languages	**0.985**	0.302	**0.966**	0.060
Bordering languages/km	**0.874**	**0.742**	**0.986**	**0.981**
Vehicularity	**0.564**	0.019	**0.595**	0.023
Roughness	**0.830**	0.069	**0.993**	**0.762**
Altitude	**1.000**	**1.000**	**1.000**	**1.000**
Small family	**0.557**	0.017	**0.794**	0.057

Results reported in columns 2, 4 are based on the incorrect method of ref. 1 (Table 2). Results reported in columns 3, 5 are computed using the BIC as PMP as justified in ref. 3. PIPs > 0.5 are highlighted in bold.

Second, to account for phylogenetic and geographic relationships, (1) use Eigenvector Spatial Filtering (ESF) (5), but their implementation has three major flaws. i) (1) select eigenvectors by minimizing residual autocorrelation (Moran’s coefficient, MC), but in ref. 5, MC is used only to identify candidate vectors; actual selection is based on model fit. The spfilteR package (6), used by (1), explicitly recommends this, noting that MC-based selection has not been established for nonlinear models. ii) To measure MC for probit models, (1) use the residual specification from ref. 7, but not in the test statistic established there. Instead, the spfilteR code applies an alternative test with a different, but unjustified normalization factor (7). Since both the residual specification and the normalization factor are model-dependent (7), the approach used by ref. 1 is invalid. iii) To account for phylogenetic and spatial interdependence, (1) use three input matrices and sum their MC values. This is incorrect, as it ignores covariation between matrices (8). A proper multimatrix extension of ref. 7 is provided in ref. 8.

The single eigenvector selected by the incorrect ESF method in ref. 1 thus fails to properly control for phylogenetic and geographic structure. Table 2 demonstrates this: We fit mixed-effects logistic regressions predicting whether a language is polysynthetic. Only models without random effects (A, B) reproduce the direction and significance of effects reported in ref. 1. All models with random intercepts for subregion and language family (C-H) fit the data far better than the best models in ref. 1, with ΔBIC > 860 in all cases (3). Computation of variance explained (9, 10) indicates that most structure is due to phylogenetic and geographic clustering. The fixed effects selected by ref. 1 are rendered insignificant or even reverse direction.

**Table 2.** Logistic regression results for predictors for polysynthetic languages and the extended list selected by ref. 1

	A	B	C	D	E	F	G	H
	Polysynth.	Extended	Polysynth.	Extended	Polysynth.	Extended	Polysynth.	Extended
Fixed effects								
L1.pop	−0.503*** (0.053)	−0.485*** (0.05)	0.118 (0.084)	0.17* (0.081)	0.434** (0.163)	0.451** (0.147)	0.322 (0.165)	0.382** (0.146)
Area	0.206* (0.094)	0.176* (0.087)	−0.129 (0.141)	−0.314* (0.13)	−0.373 (0.281)	−0.684** (0.237)	−0.543 (0.285)	−0.706** (0.221)
Bordering languages	−0.244* (0.101)	−0.201* (0.094)	−0.081 (0.13)	0.029 (0.122)	−0.056 (0.218)	0.187 (0.187)	0.063 (0.222)	0.223 (0.185)
Bordering languages/km	−0.308** (0.113)	−0.388*** (0.107)	0.158 (0.161)	−0.015 (0.154)	0.58* (0.264)	0.162 (0.227)	0.399 (0.26)	0.148 (0.224)
Vehicularity	−0.549 (0.47)	−0.582 (0.431)	−0.942 (0.541)	−0.951 (0.502)	0.182 (0.867)	0.152 (0.714)	−0.028 (0.851)	0.02 (0.713)
Roughness	−0.142 (0.079)	−0.22** (0.073)	−0.262* (0.125)	−0.278* (0.118)	−0.512* (0.254)	−0.182 (0.21)	−0.249 (0.27)	0.012 (0.228)
Altitude	0.414*** (0.068)	0.436*** (0.065)	0.173 (0.106)	0.138 (0.101)	0.317* (0.143)	0.216 (0.133)	0.061 (0.166)	−0.023 (0.155)
Small family	0.085 (0.224)	0.306 (0.199)	−0.352 (0.268)	−0.133 (0.24)	0.516 (0.597)	0.471 (0.543)	0.391 (0.604)	0.415 (0.547)
Random effects	None	None	Subregion	Subregion	Language family	Language family	Subregion × Language family	Subregion × Language family
ΔBIC	−101.659	−147.17	860.727	912.843	1,696.772	1,643.567	1,717.3	1,667.354
*R* ^2^ *_m_*	3.7%	4.3%	1.0%	1.2%	0.1%	0.1%	0.1%	0.1%
*R* ^2^_c_	n/a	n/a	75.5%	72.5%	99.5%	99.4%	99.6%	99.5%

All models are logistic regressions predicting whether a language is polysynthetic. Models A and B include only the predictors selected by ref. 1 as fixed effects. Models C to H additionally include random intercepts: subregion (C, D), language family (E, F), or subregion crossed with language family (G, H). Each cell reports the estimated coefficient (SE). Significance levels: ***P < 0.001; **P < 0.01; *P < 0.05. The final three rows report model fit metrics: ΔBIC shows the difference in BIC between each model and the best model reported in ref. 1 and *SI Appendix*, Tables S3 and S4. Positive values indicate a better fit; negative values indicate a worse fit. A |ΔBIC| greater than 10 is considered very strong evidence in favor of the model with the lower BIC (3). *R*^2^*_m_* indicates the percentage of variance explained by fixed effects only; *R*^2^_c_ includes both fixed and random effects. *P*-values are not adjusted for multiple testing.

## Data Availability

All R code needed to reproduce our results is available at https://osf.io/s5g4z/ (11). We used the data made available by ref. 1. We also supply a Microsoft Excel (xlsx) file for the calculations underlying Table 1. Additionally, data on language family was taken from Ethnologue Global Dataset, 20th edn. under a Personal Research License. To verify our results for researchers without access to the Ethnologue data, we are also using publicly available data from Glottolog via the lingtypology R package for 6,347 of 6,488 languages. Results remain qualitatively largely identical, but ΔBIC cannot be computed due to missing cases in the Glottolog data.
